# De Garengeot’s Hernia: Report of a Rare Surgical Emergency and Review of the Literature

**DOI:** 10.3389/fsurg.2018.00012

**Published:** 2018-02-16

**Authors:** Evangelos P. Misiakos, Anna Paspala, Anastasia Prodromidou, Nikolaos Machairas, Vasileia Domi, Nikolaos Koliakos, Theodore Karatzas, Nick Zavras, Anastasios Machairas

**Affiliations:** ^1^3^rd^ Department of Surgery, Attikon University Hospital, National and Kapodistrian University of Athens, Medical School, Athens, Greece; ^2^2^nd^ Department of Propaedeutic Surgery, Laiko Hospital, National and Kapodistrian University of Athens, Medical School, Athens, Greece

**Keywords:** femoral ring, hernia repair, appendicitis, appendectomy, de Garengeot's hernia

## Abstract

This is a report of a case who was admitted and operated on for a strangulated femoral hernia. The hernia sac contained a gangrenous appendix, which was excised and the hernia was repaired with sutures without complication. De Garengeot's hernia, although very rare, should be included in the differential diagnosis of cases with strangulated hernia and should receive the optimal treatment.

## Background

Femoral hernia occurs as the result of protrusion of the sac through the femoral canal medial to the femoral artery and below the inguinal ligament. This type of hernia is more common in women and accounts for only 3% of all the hernias; it has a 15–20% chance of strangulation because of the narrow and rigid femoral neck. (1–3) In only 0.5–5% of the events, the appendix can travel through the femoral hernia. Rene Jacques Croissant de Garengeot, a French surgeon, was the first to describe the presence of the vermiform appendix inside an incarcerated femoral hernia in 1731 (4). Finding de Garengeot’s hernia is rare (less than 1% of surgically treated hernias), and it is even rarer to find an acutely inflamed or perforated appendicitis within the hernia sac (roughly 0.08–0.13%) (2).

We herein report a case of de Garengeot’s hernia in a middle-aged male patient and present a systematic review of the literature.

## Case Presentation

**Figure 1:**
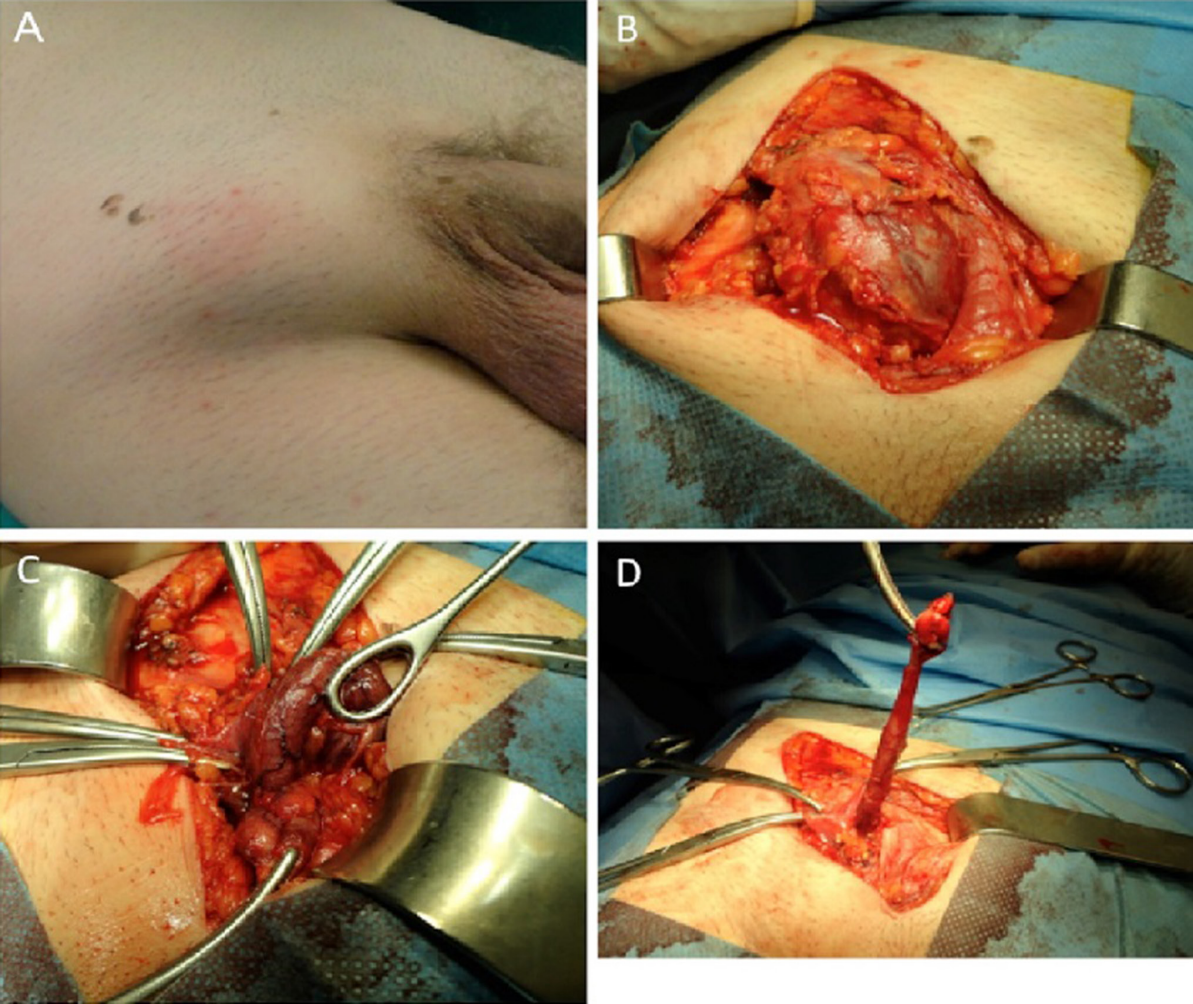
Intraoperative findings. **(A)** Irreducible lump in the right groin with overlying erythema. **(B)** Femoral sac possibly containing a gangrenous viscous. **(C)** Vermiform appendix protruding through the hernia neck with signs of advanced inflammation and ischemia. **(D)** Inflamed skeletonized appendix after ligation of the appendiceal artery.

A 56 year old male Caucasian patient was admitted to the Emergency Department of our Hospital due to an irreducible lump in his right groin, which he had initially noticed 12 h earlier. At the time of his arrival the patient had no significant abdominal pain during the last 48 h or any change in his bowel movements. Moreover, he had no personal history of hernia or any other pathology. Physical examination revealed a small irreducible palpable lump with overlying skin erythema and local tenderness in the right inguinal region (Figure 1A)*.* His abdomen was soft, non-distended, and non-tender with normal bowel sounds on auscultation and no signs of bowel obstruction. A small increase of inflammatory markers was noted in his blood tests (WBC = 13,910 × 10^9^/L; CRP = 43.8 mg/L). (Table 1) Due to gradually increasing pain in the region, the patient was transferred to the operating theater. Initially a right inguinal incision was performed. No inguinal hernia was found and a lump emerging from the subcutaneous tissue below the inguinal ligament was identified (Figure 1B)*.* After identification of the sac, the adjacent tissues were dissected, and the sac was opened. Unexpectedly the sac contained a vermiform appendix (Figure 1C) emerging from the femoral canal along with a small quantity of clear fluid (negative for bacteria). The appendix was incarcerated within the sac, inflamed and its blind end exhibited early signs of necrosis (Figure 1D). The appendix was resected and the femoral ring was approximated with sutures without use of a mesh. The patient’s postoperative course was uneventful; he tolerated oral intake and his bowel movements returned to normal within 24 h. He was discharged on the 3 postoperative day. Histology of the resected appendix showed inflammatory changes within the appendix consistent with appendicitis and peri-appendicitis.

## Discussion

Femoral hernia cases constitute an uncommon cause of groin lumps, which account for 3–5% of all abdominal hernias. The appendix is reported to be present inside the hernia sac in approximately 1% of the cases (3), and the incidence of appendicitis is even rarer, occurring in 0.08–0.13% of all patients. The clinical preoperative diagnosis of de Garengeot’s hernia can be challenging and often encountered randomly during surgery especially in cases where the patients are urgently led to surgery without preoperative imaging examination. Due to the narrow and rigid femoral neck of femoral canal, this type of hernia is much more likely to become incarcerated and strangulated. Sequentially strangulation can result in acute appendicitis or even worse in perforation and abscess formation. The treatment of choice for this type of hernia is emergency surgery. Appendectomy and primary hernia repair should be performed simultaneously.

After the year 2000, a total of 32 articles, which presented 34 cases of de Garengeot’s hernia and histology proven appendicitis have been published (1, 3, 5–34). Thirty-four patients (5 men and 29 women) with a mean age of 71.2 years with this uncommon type of hernia were presented in these studies. In the majority of cases (26/34, 76.4%) patients presented with pain in the groin or generalized pain in the lower abdomen, more frequently right-sided (only one patient had a left-sided hernia) (7). Patients reported abdominal pain, nausea and vomiting in 5 (14.7%) cases (5, 12, 13, 22, 27) and fever (8.8%) in 3 cases (6, 24, 29). Six patients underwent preoperative ultrasound in addition to a CT (13, 17, 18, 21, 30, 32) and fifteen patients had a preoperative CT as the only imaging method (1, 5, 9, 10, 12, 14, 19, 20, 22, 24–26, 29, 34). In 9 cases no imaging studies were performed as physical examination indicated an incarcerated hernia (inguinal versus femoral) and received emergency surgical treatment (3, 6, 7, 11, 23, 27, 28, 33). Seven of them had a perforated appendix (9, 11, 19, 22, 25, 28, 29). The degree of inflammation of the appendix was proven histologically in all 34 cases. Interestingly in one of these studies, Phillips et al. had described a case of a 73-year-old female who apart from appendicitis, his femoral sac included a perforated Meckel’s diverticulum (Littre’s hernia) (9).

Several surgical approaches for the treatment of de Garengeot’s hernia have been described; open (inguinal or midline incision) or laparoscopic appendectomy plus primary repair of the femoral hernia with/without mesh (Lichtenstein or TAPP technique) (3, 5, 8, 17, 20, 22, 26, 27, 30). Most surgical strategies began with an inguinal or an oblique incision over the irreducible lump (26 of the patients). Six patients had a laparotomy with a lower midline incision, because of a high possibility of abscess or perforation (9, 11, 12, 14, 16, 19). Only in 2 published cases the surgical team chose the laparoscopic approach for both appendectomy and hernia repair (one TAPP procedure and one case primary repair). (8, 22) There is currently no formal consensus regarding the optimal approach (open or laparoscopic) for the treatment of femoral hernia. Although it would be preferable not to use a mesh in a patient with well-documented inflammation, successful repair with mesh has been reported. (3, 5, 8, 17, 20, 22, 26, 27, 30) Therefore, the decision on whether to use a mesh or not depends on the surgeon’s preference in each individual case.

Herein we described a case of a 56 year old male patient, who was admitted to our center and finally underwent emergency surgery for an irreducible lump in his right groin. Intraoperatively, a de Garengeot’s hernia was identified. Pathological examination demonstrated acute appendicitis with transmural necrosis and peri-appendicitis. De Garengeot’s hernia should be in included in the physician’s differential diagnosis in patients with pain and swelling in their right groin. To that end, despite the urgency of this surgical case, surgical teams must perform imaging studies (preferably computerized tomography), which will demonstrate the exact kind of hernia (inguinal or femoral) and the content of the sac (omentum, bowel, appendix etc), with the aim to tailor the optimal surgical approach for each case.

**Table 1:** Patient characteristics

Total patients	34
Mean age ± SD (years)	71.2 ± 16.2
Gender	
*Male*	5
*Female*	29
Diagnosis	
*Preoperative* *(CT or U/S)*	25
*Intraoperative*	9
Primary symptoms	
*Pain*	26
*Nausea/Vomit*	5
*Fever*	3
Intraoperative Findings	
*Acute Appendicitis*	34
*Perforation*	7
Surgical Approach	
*Open*	32
*Laparoscopic*	2
Mesh placement	
*Yes*	9
*No*	25

## Informed Consent

Written informed consent was obtained from the patient for the publication of this case report.

## Author Contributions

EM is the chief surgeon of this case and the main author of this case report, APa helped him in this operation and, with VD, contributed in the collection and interpretation of data. NZ is a pediatric Surgeon in our Department and contributed in analyzing the patient’s clinical reports and obtained informed consent of the patient. NK and NM are chief resident and postdocroral fellow, respectively, in our Department and helped in reference collection and selection, and the writing of the paper. APr and TK are surgeons from another Hospital who helped in the collection of literature references and review analysis. AM is the Professor and Chairman in our Department and supervised the writing of this report.

## Conflict of Interest Statement

The authors declare that the research was conducted in the absence of any commercial or financial relationships that could be construed as a potential conflict of interest.

## References

[1] Kagan Coskun A Kilbas Z Yigit T Simsek A Harlak A De Garengeot's hernia: the importance of early diagnosis and its complications. *Hernia* (2012) 16(6):731–3. 10.1007/s10029-011-0814-0 21431837

[2] Kalles V Mekras A Mekras D Papapanagiotou I Al-Harethee W Sotiropoulos G De Garengeot's hernia: a comprehensive review. *Hernia* (2013) 17(2):177–82. 10.1007/s10029-012-0993-3 22983696

[3] Konofaos P Spartalis E Smirnis A Kontzoglou K Kouraklis G De Garengeot's hernia in a 60-year-old woman: a case report. *J Med Case Rep* (2011) 5:258 10.1186/1752-1947-5-258 21718485 PMC3141709

[4] De Garengeot RJC *Traite des operations de chirurgie*. 2nd ed Paris: Huart (1731). p. 369–71.

[5] Ebisawa K Yamazaki S Kimura Y Kashio M Kurito K Yasumuro S Acute appendicitis in an incarcerated femoral hernia: a case of De Garengeot hernia. *Case Rep Gastroenterol* (2009) 3(3):313–7. 10.1159/000250821 21103247 PMC2988923

[6] Piperos T Kalles V Al Ahwal Y Konstantinou E Skarpas G Mariolis-Sapsakos T Clinical significance of de Garengeot's hernia: a case of acute appendicitis and review of the literature. *Int J Surg Case Rep* (2012) 3(3):116–7. 10.1016/j.ijscr.2011.12.003 22288062 PMC3267278

[7] Caygill P Nair R Sajjanshetty M Francis D An unusual groin exploration: De Garengeot's hernia. *Int J Surg Case Rep* (2011) 2(5):74–5. 10.1016/j.ijscr.2011.01.008 22096687 PMC3199633

[8] Comman A Gaetzschmann P Hanner T Behrend M DeGarengeot hernia: transabdominal preperitoneal hernia repair and appendectomy. *JSLS* (2007) 11(4):496–501. 18237518 PMC3015839

[9] Phillips AW Aspinall SR Appendicitis and Meckel's diverticulum in a femoral hernia: simultaneous De Garengeot and Littre's hernia. *Hernia* (2012) 16(6):727–9. 10.1007/s10029-011-0812-2 21442431

[10] Ryan JW O'Riordan I Gorey T Geoghegan T de Garengeot hernia with a mucinous neoplasm of the appendix, two clinical rarities combine to yield a first for the literature. *BMJ Case Rep* (2017) 2017:pii: bcr-2017-220830 10.1136/bcr-2017-220830 PMC5535124 28611141

[11] Nguyen ET Komenaka IK Strangulated femoral hernia containing a perforated appendix. *Can J Surg* (2004) 47(1):68–9. 14997930 PMC3211803

[12] Hsiao TF Chou YH Appendiceal pus in a hernia sac simulating strangulated femoral hernia: a case report. *Int J Gen Med* (2011) 4:235–7. 10.2147/IJGM.S16413 21556351 PMC3085233

[13] Brown N Moesbergen T Steinke K The French and their hernias: prospective radiological differentiation of de Garengeot from other groin hernias. *J Radiol Case Rep* (2013) 7(4):16–21. 10.3941/jrcr.v7i4.831 PMC3661430 23705048

[14] Shah A Sira Janardhan H De garengeot hernia: a case report and review of literature. *Indian J Surg* (2013) 75(Suppl 1):439–41. 10.1007/s12262-012-0778-z 24426642 PMC3693382

[15] Hussain A Slesser AA Monib S Maalo J Soskin M Arbuckle J A De Garengeot hernia masquerading as a strangulated femoral hernia. *Int J Surg Case Rep* (2014) 5(10):656–8. 10.1016/j.ijscr.2014.08.001 25194597 PMC4189075

[16] Madiha A Rares H Abdus S De Garengeot hernia: a forgotten rare entity? *BMJ Case Rep* (2014) 2014:pii: bcr2013201413:bcr2013201413 10.1136/bcr-2013-201413 PMC3987227 24722706

[17] Ramsingh J Ali A Cameron C Al-Ani A Hodnett R Chorushyj C De Garengeot's hernia: diagnosis and surgical management of a rare type of femoral herniapii: rju008. *J Surg Case Rep* (2014) 2014(2):rju008 10.1093/jscr/rju008 PMC4164193 24876373

[18] Schäfer HM von Holzen U Nebiker C Swelling of the right thigh for over 30 years-The rare finding of a De Garengeot hernia. *Int J Surg Case Rep* (2014) 5(12):1120–2. 10.1016/j.ijscr.2014.11.010 25437653 PMC4275792

[19] Ahmed K Bashar K Mchugh TJ Mchugh SM Kavanagh E Appendicitis in De Garengeot's hernia presenting as a nontender inguinal mass: case report and review of the literature. *Case Rep Surg* (2014) 2014:932638 10.1155/2014/932638 24716081 PMC3971513

[20] Liipo TK Seppälä TT Mattila AK De Garengeot's hernia: 40 years after Bassini inguinal hernioplasty. *BMJ Case Rep* (2015) 2015:bcr2014208327 10.1136/bcr-2014-208327 PMC4369007 25733091

[21] Pan CW Tsao MJ Su MS A case of De Garengeot hernia requiring early surgery. *BMJ Case Rep* (2015) 2015:bcr2015211102 10.1136/bcr-2015-211102 PMC4513554 26199302

[22] Garcia-Amador C de La Plaza R Arteaga V Lopez-Marcano A Ramia J Garengeot's hernia: two case reports with CT diagnosis and literature review. *Open Med* (2016) 11(1):354–60. 10.1515/med-2016-0065 PMC5329852 28352820

[23] Sinraj AP Anekal N Rathnakar SK De Garengeot's hernia - a diagnostic and therapeutic challenge. *J Clin Diagn Res* (2016) 10(11):PD19–20. 10.7860/JCDR/2016/21522.8871 PMC5198391 28050438

[24] Bidarmaghz B Tee CL A case of De Garengeot hernia and literature review. *BMJ Case Rep* (2017) 2017:bcr-2017-220926 10.1136/bcr-2017-220926 PMC5589054 28882935

[25] Bloom A Baio FE Kim K Fernandez-Moure JS Reader M Diagnosis and operative management of a perforated de Garengeot hernia. *Int J Surg Case Rep* (2017) 41:114–6. 10.1016/j.ijscr.2017.10.009 29059610 PMC5651548

[26] Shiihara M Kato T Kaneko Y Yoshitoshi K Ota T de Garengeot hernia with appendicitis treated by two-way-approach surgery: a case report. *J Surg Case Rep* (2017) 2017(7):rjx140 10.1093/jscr/rjx140 28775838 PMC5534011

[27] González Alcolea N Martínez Arrieta F Lucena de La Poza JL Jiménez Cubedo E Sánchez Turrión V De Garengeot's hernia: incarcerated femoral hernia containing the vermiform appendix. Report of two cases and literature review. *Cir Esp* (2017) 95(3):177–8. 10.1016/j.ciresp.2016.08.005 27865427

[28] Taveras LR Huerta S A case report of a de Garengeot hernia in a nonagenarian veteran. *Int J Surg Case Rep* (2017) 41:301–3. 10.1016/j.ijscr.2017.10.049 29127919 PMC5683743

[29] Georgiou GK Bali C Theodorou SJ Zioga A Fatouros M Appendiceal diverticulitis in a femoral hernia causing necrotizing fasciitis of the right inguinal region: report of a unique case. *Hernia* (2013) 17(1):125–8. 10.1007/s10029-011-0822-0 21541716

[30] Hao J Yao J Guo D Sun W Liang J Jiang X De Garengeot hernia: the ultrasound and computed tomographic findings in an 81-year-old woman. *Am J Emerg Med* (2014) 32(5):486.e5–6. 10.1016/j.ajem.2013.11.003 24332902

[31] Rajan SS Girn HR Ainslie WG Inflamed appendix in a femoral hernial sac: de Garengeot's hernia. *Hernia* (2009) 13(5):551–3. 10.1007/s10029-009-0472-7 19225856

[32] Ring A Gelis V Klupsch C Stern J De Garengeot appendicitis - rare variant of a common medical condition. *Zentralbl Chir* (2009) 134(6):564–6. 10.1055/s-0029-1224564 20020391

[33] Suppiah A Barandiaran J Morgan R Perry EP First case of villous adenoma of the appendix leading to acute appendicitis presenting as strangulated femoral hernia: changes in management owing to concurrent adenoma. *Hernia* (2008) 12(1):95–8. 10.1007/s10029-007-0245-0 17566835

[34] Tanrıkulu CS Tanrıkulu Y Akkapulu N De Garengeot's hernia: a case of acute appendicitis in a femoral hernia sac. *Ulus Travma Acil Cerrahi Derg* (2013) 19(4):380–2. 10.5505/tjtes.2013.37043 23884684

